# Unmet financial obligations and alcohol-related mortality: A nationwide register-based follow-up study

**DOI:** 10.1016/j.ssmph.2022.101139

**Published:** 2022-06-13

**Authors:** Yerko Rojas

**Affiliations:** School of Social Sciences, Södertörn University, SE-141 89, Huddinge, Sweden

**Keywords:** Debt problem, Over-indebtedness, Enforcement authority, Alcohol-related harm, Stressful life event, Sweden

## Abstract

This study sets out to explore whether experiencing financial indebtedness is related to alcohol-related mortality. For this purpose, people aged between 20 and 64 having a registration date for a debt in the Swedish Enforcement Authority's register during 2015 (n = 48,541) were followed up for a five-year period for alcohol-related mortality and were compared with a sample from the general Swedish population (n = 261,148). On the basis of logistic regression analysis, it is shown that people who had experienced financial indebtedness were almost two and a half times more likely to suffer from alcohol-related death than those who had not lived through this experience (OR = 2.43), controlling for several demographic, socio-economic, and health conditions prior to the date of the registration at the Enforcement Authority. The results provide support for the notion that debt repayment problems may, in itself, be an important indicator to consider in the study of alcohol-related harm. Consequently, debt counselling and other programs directed toward mitigating debt-related stress may play an important role in alleviating the adverse effects of indebtedness.

## 1 Introduction

The predominant theoretical rationale underlying the expected relation between financial strain and alcohol use, commonly referred to as the tension-reduction hypothesis, assumes that people use alcohol to relieve the negative emotions resulting from stress exposure (Shaw, Agahi, & Krause, 2011). However, not much is known about whether alcohol-related outcomes can be traced back to actually having failed to meet one's financial obligations (cf., Collins, 2016; Turunen & Hiilamo, 2014). This is despite the fact that a standard definition of financial strain is the degree to which people perceive that their financial demands surpass their capability to meet those demands (Serido, Lawry, Li, Conger, & Russell, 2014), and that economic problem in general is a key conceptual component in the well-established literature showing that low socio-economic status is related to a wide array of alcohol-related harm (death included). This knowledge gap is quite problematic, partly because there are indications that debts and problem drinking may be related to each other (Jenkins et al., 2008; Richardson, Elliott, & Roberts, 2013), partly because the current transition toward a much more debt-based economy has resulted in increasing debt repayment problems, in particular among the most socio-economically vulnerable in the society (Callegari, Liedgren, & Kullberg, 2019; Lazzarato, 2012).

The need to identify specific economic causes for alcohol-related outcomes has been stressed previously, particularly in discussions on how to better tackle the so-called alcohol-harm paradox (Boyd et al., 2021). This paradox being the known public health occurrence that disadvantaged groups suffer from higher rates of alcohol-related hospital admissions and deaths compared to advantaged groups, despite reporting similar or lower average levels of consumption (Bloomfield, 2020; Boyd et al., 2021; Ponnet, 2014). Trouble paying one's bills and problems with creditors are typical indicators of economic problems in adjacent academic disciplines (e.g., Agnew, Matthews, Bucher, Welcher, & Keyes, 2008; Ponnet, 2014). They not only have the potential to capture a considerable change in lifestyle due to lack of money and the inability to buy needed goods and services, but also the actual or threatened loss of valued services and goods as well as an actual or threatened presentation of negative stimuli that are a direct result of lack of money (Agnew, 1992, 1994; Agnew et al., 2008).

This study sets out to explore whether financial indebtedness, understood as having a registration date for a debt in the register at the Swedish Enforcement Authority (SEA) during 2015, among adults aged between 20 and 64 years old, is linked to alcohol-related mortality during the period 2015–2019. There are at least two important advantages for studying the relationship in this way: (a) to be registered for a debt-related issue at an enforcement authority constitutes a key stage of the indebtedness process, in terms of distress (Bond & Holkar, 2018). In Sweden, a record of non-payment makes it difficult for a person to sign for services, including a lease, an insurance policy, or a mobile subscription (SOU, 2013:78); (b) examining cases in which a non-payment of debt has been officially registered at the SEA has been described as the most reliable measure of over-indebtedness available in Sweden (SOU, 2013:78) and also falls well within what the international literature classify as the administrative model of studying financial indebtedness (Turunen & Hiilamo, 2014).

For the purpose of the study, the opportunity offered by the Microdata Online Access (MONA) system at Statistics Sweden was used to link and analyze data from different nationwide registers. Including data on debtors’ socio-economic, demographic, criminal, and health status prior to their debt problems made it possible to adjust for confounding factors and generalize the findings in an unprecedented manner. The study also employs a comparison group – matched by age, gender, and region of residence – comprising a sample of the Swedish population.

## 2 Method

### 2.1 Cases: the Swedish Enforcement Authority (SEA) register

The SEA is a government agency with a range of responsibilities, including, but not limited to, debt collection, enforcement, and injunctions to pay (cf., Kronofogden, 2020; Regeringskansliet, 2021). Anybody with a legitimate payment claim can turn to the SEA, for example, the government, municipalities, companies, and private individuals (Kronofogden, 2020; Regeringskansliet, 2021). The SEA registers all debts that have been confirmed through a court order or a simplified and accelerated procedure for order of payment carried out by the SEA itself and in respect of which, the debtor has failed to pay the debt (Jørgensen, 2016).

The exposed group in this study includes all adults aged 20–64 years (at baseline), including both women and men, with a registration date for debt at the SEA (Kronofogden) register between January 1, 2015 and December 31, 2015. The information from the SEA's database was extracted on January 11, 2018. An individual who has been registered at the SEA may be kept in the register for five years but can be removed earlier. In practice, a debtor is kept in the register for a minimum of three years after his or her latest matter has been closed by the SEA (SOU, 2013:78). This means that the cases in the exposed group share the experience of not having been active for an unpaid debt at the SEA in the recent past, prior to 2015 (approximately 3 years, at least). (cf., SOU, 2013:78; Vuleta, 2018)

### 2.2 A matched comparison group: other national register data

The data from the SEA register have been linked to several other national registers. This study makes use of the linkages made with (a) the Medicinal Drug Register, the National Patient Register, and the National Cause of Death Register; (b) the register of persons convicted of criminal offences; and (c) the longitudinal integration database for health insurance and labor market studies (known in Sweden as LISA), the population statistics register, and the geography database. These registers are administered by the National Board of Health and Welfare, the Swedish National Council for Crime Prevention, and Statistics Sweden, respectively.

The comparison group was created by Statistics Sweden. For each of the individuals registered at the SEA (i.e., for each of the individuals in the exposed group), a set of up to five controls was extracted (December 31, 2014) from the general Swedish population, matched by age, gender, and region of residence. The data set for the comparison group comprised the same information from the national registers as the data set for the exposed group. The individuals registered at the SEA were removed from the sample population before the comparison group was created.

The research project has been approved by the Swedish Ethical Review Authority (reference number: 2017/2227-31/5). All the data are stored at Statistics Sweden and have been made available to the author via its micro-data Online Access (MONA) system.

### 2.3 Analytical strategy

The available follow-up information on cause of death for those registered for a debt at the SEA in 2015 is limited to the years 2015–2019, that is, to a follow-up period of 4–5 years. Consequently, the analysis of the over-indebted population is restricted to alcohol-related death occurring within five years of the registration date at any point during the period 2015. The comparison with the matched sample of the Swedish population is restricted to the same 5-year follow-up period; more precisely, to alcohol-related mortality occurring within 5 years after the sample was drawn (December 31, 2014). The control variables are measured at baseline for both the exposed and the comparison group. The demographic, criminal, and socio-economic status variables are measured during the calendar year preceding the start of the follow-up period. The other control variables, all indicators of health status (mood disorder, in-patient care for substance abuse, in-patient care for diseases of the digestive system, and sickness cash benefit recipients), are measured throughout the three calendar years preceding the start of the follow-up period.

The length of the follow-up period for measuring both alcohol-related death (5 years) and the control variables (1–3 years, respectively) is a design that is in line with how an exposure to a stressful life event (job loss) and alcohol-related mortality has been studied previously (Eliason, 2014).

Personal identification numbers identified as erroneous have been excluded, as well as those who emigrated during the study period. The analysis only considers people for whom complete data are available for all the variables included in the models. However, for alcohol-related death cases that only contained information on the year and month of the date of death, the first day of the month was assigned as the day of death.

The relationship between the independent/control variables and alcohol-related death has been estimated using logistic regression. The loose-matching nature of the data under study allows for an analysis using unconditional logistic regressions (Kuo, Duan, & Grady, 2018). The advantage of this is that it is possible to obtain estimates for the matching variables by simply including them as regular control variables in the analysis.

Given the difficulty in merely relying on P-values (and significance tests based on them) for statistical inference when using large-sample data (Raftery, 1995), as is the case in this study, the Bayesian Information Criterion (BIC) value was used as a complementary statistical tool for the selection of the model. BIC is widely used as a variable-selection criterion, partly because its penalty term is a product of the number of parameters in the model and the log of the sample. (StataCorp, 2021). When comparing models, the model with the smallest BIC-value is preferred. A minimum BIC difference of 2.00 was the critical value employed to conclude that the model including more control variables was more appropriate than the one including fewer control variables (Raftery, 1995).

### 2.4 Dependent variable

Alcohol-related mortality is defined as deaths with alcohol-related diagnoses written on the death certificate – either as an underlying or a contributory cause of death – and registered in the Swedish Cause of Death Register at any point during the follow-up period. It is a dichotomous variable, coded as 1 if the individual was found in the Cause of Death Register with a registration of an alcohol-related death between 2015 and 2019, and as 0 otherwise. This way of measuring alcohol-related mortality can be found in previous research (e.g., Eliason, 2014; Hemström, 2002; Herttua, Mäkelä, & Martikainen, 2008), as well as in official national statistics (Folkhälsomyndigheten, 2022).

For a detailed description of the alcohol-related diagnoses that the National Board of Health and Welfare (Socialstyrelsen, 2016) recommend for the healthcare system in Sweden (out-patient and in-patient care) to use when classifying use and abuse of alcohol at a national, regional, and local level, in accordance with the Swedish version of the 10th revision of the International Classification of Diseases (ICD-10-SE), please see Table A.1, appendix A.

**Table 1:** Distribution of dependent and control variables included in the models by the group that had a registration date for a debt at the Swedish Enforcement Authority during 2015 (exposed group) and the matched sample of the Swedish population (comparison group).

Variable	Respondents	
	Exposed group	Comparison group
	(n = 48,514)	(n = 261,122)
Alcohol-related death (%)	0.20	0.04
*Control variables – measured in 2014*		
Place of birth (%)		
Born in Sweden (reference category: foreign born)	72.35	83.59
Single-person household (%)		
Single (reference category: other family constellations)	23.76	18.07
Disposable income (Mean)		
Personal disposable income (centile) [100 SEK]	4.79 [1871.4]	5.78 [2197.7]
Unemployment (%)		
Unemployed (reference category: other)	20.60	9.81
Social welfare recipiency (%)		
Received social assistance (reference category: other)	11.42	2.97
Education (%)		
Pre-upper secondary (reference category: post-upper secondary education)	20.56	9.92
Upper secondary (reference category: post-secondary education)	51.68	49.24
Criminality (%)		
Convicted of a criminal offence (reference category: other)	3.76	0.71
Age (Mean)		
Year of birth	1976.84	1976.72
Gender (%)		
Men (reference category: women)	60.32	61.17
Region of residence (%)		
Living in a big city (reference category: other)	56.53	57.16
*Control variables – measured in 2012–2014*		
Ill-health (%)		
Received sickness cash benefit (reference category: other)	20.75	17.08
Mood disorder (reference category: other)	19.59	10.81
Mental and behavioral disorders due to psychoactive substance use (reference category: other)	1.92	0.33
Digestive diseases (reference category: other)	2.44	1.78

### 2.5 Exposure variable

Financial indebtedness is defined as having a registration date for a debt in the SEA register at any point between January 1, 2015 and December 31, 2015 (yes = 1, otherwise = 0).

### 2.6 Control variables

Although knowledge concerning the relationship between financial indebtedness and alcohol-related harm is limited (cf., Collins, 2016; Turunen & Hiilamo, 2014), there is more knowledge about the factors that may be important to control for, in order to assess the extent to which a stressful life event, in itself, can be viewed as a risk factor for alcohol-related mortality (Eliason, 2014). In this case, controlling for well-established risk factors, known to be linked to both registration at the SEA for a debt and alcohol-related mortality, such as criminality, depression, substance abuse, sickness, welfare recipiency unemployment, income, and low education (Collins, 2016; Eliason, 2014; Kronofogden, 2008; SOU, 2013:78; Stenbacka, Moberg, & Jokinen, 2019; Vuleta, 2018), is of special importance.

The control variables have been measured in accordance with the analytical strategy described above and are defined as follows: *Gender:* women/men; *Age:* year of birth; *Region of residence*: living in one of the three regions in Sweden that includes the country's three largest cities (Stockholm, Gothenburg, and Malmö, respectively); *Place of birth:* foreign born/born in Sweden; *Family status*: single persons/all types of family constellations (married families [including civil unions], cohabiting families, and one-parent families); *Education*: pre-upper-secondary, upper-secondary, and post-upper-secondary education; *Disposable income:* income deciles. The income decile categorization is based on the personal disposable income distribution of the comparison group. The first income decile contains the lowest income tenth, and the last one contains the highest income tenth. Other control variables include *Unemployment:* being registered as unemployed at the relevant authorities for at least one day over the course of a 1-year period; and *Social welfare recipiency:* having received means-tested social assistance at least once over the course of a year. Finally, *Ill-health* is measured using four different indicators: *Mood disorder:* registered as having been prescribed anti-depressants (ATC-code: N06A) at least once over a 3-year period; *Substance abuse:* been recorded in the Swedish in-patient care register with mental or behavioral disorders due to psychoactive substance use as a main diagnosis (ICD-10: F10–F19) at least once over a 3-year period; *Sickness cash benefit:* received sickness cash benefits from the Swedish Social Insurance Agency at least once (measured in terms of net days) over a 3-year period; and *Digestive diseases:* been recorded in the Swedish in-patient care register with diseases of the digestive system as a main diagnosis (ICD-10: K00-93) at least once over a 3-year period.

## 3 Results

The study base consists of an exposed group and a comparison group, comprising a total of 48,541 and 261,148 individuals, respectively (see Table 1). A total of 196 alcohol-related deaths are included in the analysis, of which 96 occurred in the exposed group and 100 in the comparison group (see Table 1). The proportion of people who died from an alcohol-related death in the exposed group is approximately five times as large as the corresponding proportion in the comparison group (see Table 1). Apart from the matching variables of age, gender, and region of residence, the distributions of the control variables clearly differ between the exposed group and the comparison group, confirming the adverse conditions of those people who experienced financial indebtedness (see Table 1).

The results from the logistic regression analysis are presented in Table 2. In Model 1, it is shown that financial indebtedness is significantly related to alcohol-related mortality, with an OR of 5.17. In other words, people who had a registration date for a debt at the SEA register at any point between January 1, 2015 and December 31, 2015 were approximately five times more likely to have an alcohol-related death than people who had not been exposed to this experience. As can be seen in Model 2, this relationship is somewhat weakened when adjusted for age, gender, place of birth, region of residence, and single-person household (cf. OR = 5.17 with OR = 4.71). However, the data do not support the inclusion of interaction effects between financial indebtedness and the statistically significant and newly introduced control variables in its relation to alcohol-related death; none of the multiplicative models (that is, with interaction terms) produced smaller BIC-values than its additive counterpart (that is, without interaction terms). The BIC-values for the tested multiplicative models are presented in Table B.1, appendix B.

**Table 2:** Logistic regression of financial indebtedness and alcohol-related mortality among adults aged 20–64 in Sweden 2015–2019

Variable	Model 1 Crude OR (95% CI)	Model 2 Adjusted OR (95% CI)	Model 3 Adjusted OR (95% CI)	Model 4 Adjusted OR (95% CI)
*Independent variable*				
**Financial indebtedness**				
Debt registered at the Swedish Enforcement Authority (reference category: other)	5.17 (3.91–6.85)***	4.71 (3.54–6.26)***	3.04 (2.24–4.12)***	2.43 (1.77–3.32)***
*Control variables*				
Age				
Year of birth (continuous)		0.91 (0.90–0.92)***	0.90 (0.89–0.91)***	0.90 (0.89–0.91)***
Gender				
Men (reference category: women)		1.84 (1.33–2.55)***	1.95 (1.40–2.72)***	2.07 (1.47–2.90)***
Place of birth				
Born in Sweden (reference category: foreign born)		1.37 (0.93–2.02)	2.24 (1.49–3.38)***	1.84 (1.22–2.79)**
Region of residence				
Living in a big city (reference category: other)		1.03 (0.77–1.36)	1.17 (0.87–1.55)	1.12 (0.84–1.50)
Single-person household				
Single (reference category: other family constellations)		3.40 (2.55–4.51)***	2.92 (2.18–3.91)***	2.39 (1.77–3.23)***
Unemployment				
Unemployed (reference category: other)			1.56 (1.08–2.26)*	1.56 (1.07–2.27)*
Social welfare recipiency				
Received social assistance (reference category: other)			2.11 (1.35–3.29)**	1.56 (0.99–2.46)
Disposable income				
Personal disposable income (deciles)			0.84 (0.79–0.88)***	0.85 (0.80–0.90)***
Education				
Pre-upper secondary (reference category: post-upper secondary education)			2.13 (1.38–3.30)**	1.92 (1.23–2.98)**
Upper secondary (reference category: post-secondary education)			1.74 (1.18–2.57)**	1.60 (1.08–2.37)*
Criminality				
Convicted of a criminal offence (reference category: other)			3.59 (2.02–6.37)***	2.28 (1.24–4.18)**
Ill-health				
Mood disorder (reference category: other)				1.90 (1.37–2.63)***
Mental and behavioral disorders due to psychoactive substance use (reference category: other)				9.52 (6.26–14.45)***
Digestive diseases (reference category: other)				3.05 (1.90–4.88)***
Received sickness cash benefit (reference category: other)				1.08 (0.77–1.50)
Alcohol-related deaths	196	196	196	196
*The model's BIC-value*^a^	BIC = 3186.0	BIC = 2891.7	BIC = 2846.4	BIC = 2751.8
Total study population (n)	n = 309,689	n = 309,689	n = 309,689	n = 309,689

a BIC is calculated using N = number of observations. * p < 0.05, **p < 0.01 and ***p < 0.001.

When five additional control variables are introduced into the analysis (social welfare recipiency, unemployment, education, disposable income, and criminality), the effect of financial indebtedness on alcohol-related mortality remains significant but decreases in strength, from an OR of 4.71 to one of 3.04 (see Model 3 in Table 2). Once again, the data did not support the inclusion of interaction effects between financial indebtedness and the statistically significant and newly introduced control variables in its relation to alcohol-related death; none of the multiplicative models produced smaller BIC-values than its additive counterpart (cf., Table 2 with Table B.1, appendix B).

In the final model (Table 2), ill-health (measured using four different indicators) is included in the analysis. The effect of financial indebtedness decreases once again, culminating in an odds ratio of 2.43, but remains significant (see Model 4). In this model too, no support was found in the data for financial indebtedness varying with any of the statistically significant and newly introduced control variables, that is, none of the estimated multiplicative models produced smaller BIC-values than its additive counterpart (cf., Table 2 with Table B.1, appendix B).

In Table 3, the final model was replicated, excluding, one at a time, death from leading underlying causes of death in alcohol-related mortality. The objective here was to examine how sensitive the final results were to the so-called acute and chronic underlying causes of death – that is, to non-alcohol attributable diseases (e.g., alcohol poisonings) and to alcohol attributable diseases (e.g., alcoholic liver diseases and alcoholic diseases of the pancreas) – in accordance with the 10^th^ revision of the International Classification of Diseases (ICD-10). As can be seen in sensitivity test number 4 (Table 3), of the 196 alcohol-related deaths included in the analysis, 72 had an alcohol attributable disease as an underlying cause of death (ICD-10 codes: I426, F10.1, F10.2, F10.7, K70.0, K70.1, K70.3, K70.4, K85.2, K86.0) (cf., Herttua et al., 2008; Lahti & Vuori, 2002). The relationship found between financial indebtedness and alcohol-related deaths remained practically the same, both in terms of size and statistical significance (cf., Table 3 with Model 4 in Table 2).

**Table 3:** Logistic regression of financial indebtedness and alcohol-related mortality among adults aged 20–64 in Sweden 2015–2019, excluding underlying causes of death from fatal alcohol poisonings, liver disease, and diseases of the pancreas, all/non-alcohol attributable diseases, respectively.

Variable	Sensitivity test 1 (alcohol-related death, except alcohol poisonings^a^) Adjusted OR (95% CI)	Sensitivity test 2 (alcohol-related death, except liver/pancreatic diseases^b^) Adjusted OR (95% CI)	Sensitivity test 3 (alcohol-related death, except liver/pancreatic/other alcohol attributable diseases^c^) Adjusted OR (95% CI)		Sensitivity test 4 (alcohol-related death, except non-alcohol attributable diseases^d^) Adjusted OR (95% CI)
**Debt registered at the Swedish Enforcement Authority (reference category: other)**	2.50 (1.80–3.47)***	2.34 (1.64–3.34)***	2.17 (1.46–3.22)***		2.85 (1.71–4.77)***
Age					
Year of birth (continuous)	0.90 (0.89–0.91)***	0.91 (0.89–0.92)***	0.91 (0.90–0.93)***		0.88 (0.86–0.90)***
Gender					
Men (reference category: women)	1.91 (1.35–2.70)***	2.92 (1.92–4.44)***	2.55 (1.64–3.96)***		1.50 (0.89–2.53)
Place of birth					
Born in Sweden (reference category: foreign born)	1.91 (1.24–2.95)*	1.76 (1.10–2.81)*	1.58 (0.96–2.61)*		2.41 (1.17–4.95)*
Region of residence					
Living in a big city (reference category: other)	1.06 (0.78–1.43)	1.30 (0.93–1.81)		1.27 (0.88–1.83)	0.93 (0.58–1.49)
Single-person household					
Single (reference category: other family constellations)	2.39 (1.74–3.28)***	2.20 (1.56–3.09)***		2.01 (1.37–3.94)***	3.09 (1.87–5.09)***
Unemployment					
Unemployed (reference category: other)	1.50 (1.01–2.24)*	1.72 (1.14–2.59)*		1.97 (1.26–3.05)**	0.95 (0.46–1.95)
Social welfare recipiency					
Received social assistance (reference category: other)	1.62 (1.01–2.59)*	1.26 (0.74–2.15)	1.26 (0.71–2.25)		2.31 (1.15–4.66)*
Disposable income					
Personal disposable income (deciles)	0.84 (0.79–0.89)***	0.86 (0.81–0.92)***	0.87 (0.81–0.93)***		0.84 (0.76–0.92)***
Education					
Pre-upper secondary (reference category: post-upper secondary education)	2.06 (1.30–3.27)**	2.54 (1.53–4.23)***	1.60 (0.92–2.80)		2.40 (1.15–5.03)*
Upper secondary (reference category: post-secondary education)	1.59 (1.05–2.41)*	1.80 (1.13–2.86)*	1.56 (0.97–2.51)		1.68 (0.84–3.33)
Criminality					
Convicted of a criminal offence (reference category: other)	1.69 (0.85–3.42)	2.20 (1.17–4.33)*	2.34 (1.15–4.77)*		1.98 (0.64–6.06)
Ill-health					
Mood disorder (reference category: other)	1.66 (1.18–2.35)**	1.90 (1.31–2.76)*	2.03 (1.35–3.06)**		1.62 (0.95–2.75)
Mental and behavioral disorders due to psychoactive substance use (reference category: other)	9.80 (6.32–15.21)***	10.57 (6.58–16.97)***	12.53 (7.53–20.85)***		5.56 (2.70–11.46)***
Digestive diseases (reference category: other)	3.26 (2.01–5.29)***	1.26 (0.62–2.58)	1.40 (0.66–2.99)		6.39 (3.47–11.77)***
Received sickness cash benefit (reference category: other)	1.06 (0.74–1.51)	1.27 (0.88–1.85)	1.28 (0.85–1.92)		0.79 (0.44–1.41)
Alcohol-related deaths	178	151	124		72
Total study population (n)	n = 309,671	n = 309,644	n = 309,617		n = 309,565

a ICD-10 codes X45, T5.

b ICD-10 codes K70.0, K70.1, K70.3, K70.4, K85.2, K86.0.

c ICD-10 codes: I426, F10.1, F10.2, F10.7, K70.0, K70.1, K70.3, K70.4, K85.2, K86.0.

d ICD-10 codes: not alcohol attributable diseases (c). * p < 0.05, **p < 0.01 and ***p < 0.001.

## 4 Discussion

To the best of my knowledge, this study is the first to examine the relationship between financial indebtedness and alcohol-related mortality, using large-scale register data for an entire country (cf., Collins, 2016; Turunen & Hiilamo, 2014). This has made it possible to control, in an unprecedented way, for crucial factors that may have been biasing the relationship, including other economic stressors (such as unemployment, social welfare recipiency and income). The fact that (a) a total of 96 of the 196 deaths included in the analysis occurred in the exposed group; (b) the effect of financial indebtedness remained significantly related to alcohol-related mortality in a detrimental way when adjusting for relevant background variables; and (c) the found effect is comparable to the size of the effects of well-established socio-economic risk factors behind alcohol-related mortality (cf., Collins, 2016), has both important theoretical and policy implications, three of which are particularly important.

First, we need to acknowledge that the experience of being registered at an enforcement authority due to unmet financial obligations may be a major source of strain that is not reducible to the factors leading to having fallen into debt problems in the first place (Bond & Holkar, 2018). From a theoretical perspective, it could even be argued that officially registered debt repayment problems is par excellence the objective counterpart of “the extent to which individuals perceive that their financial demands exceed their ability to meet those demands” (Serido, Joyce, Charles, Conger, & Russell, 2014, p. 340), which is how the concept of financial strain has tended to be understood in the literature on alcohol. The question is whether it is not even a more precise way of capturing the tension-reduction mechanism assumed to be at work in the relationship between socio-economic factors and alcohol-related harm than many of the proxies of socio-economic status usually used to study this issue (e.g., level of education (Collins, 2016)). If this were to be the case, it could be argued that the study has found empirical support for a distinctive, relatively proximate, and dynamic economic risk factor that previous research on social inequality and alcohol-related harm have overlooked (Boyd et al., 2021; Probst, Kilian, Sanchez, Lange, & Rehm, 2020). This is an important finding, not only because it might help us to further understand the alcohol-harm paradox – after all, lower socio-economic groups are more likely to have debt problems (Kronofogden, 2008; Oksanen, Aaltonen, & Rantala, 2015) – but also because it gives further legitimacy to the developments of policies, focusing on targeting economic circumstances in the efforts to come to terms with this complex phenomenon (Boyd et al., 2021).

Second, professionals and others who interact with individuals who are in the process of falling into a situation of unmanageable debt may be important ‘gatekeepers’ in preventing alcohol-related mortality. Although the academic social work discourse has tended to neglect the issue of debt problems (cf., Callegari et al., 2019; Dellgran, 2000; Henrikson & Ingvarsson, 2019), social workers, in particular, have a unique role to play in this respect. They not only have the knowledge, attitudes, and skills to identify individuals at risk of alcohol-related problems, determining the level of risk, and then referring at-risk individuals for treatment, but, as they become increasingly involved with people with debt problems (Callegari et al., 2019), they are also in a position to identify people at risk at a very early stage. In fact, as of November 2016, the Social Services Act (SoL) (SFS, 2001:453, 5 chapter 12 §) in Sweden, which regulates social care services, includes a clause, which stipulates that municipalities are expected to provide budget and debt advice to indebted people – both to prevent financial indebtedness and to help them find a solution to their problems.

Finally, and given that there are few social problems, such as the inability to repay a debt, that have such a strong individual stigma attached to them (Di Leo, Hitchcock, & McClennen, 2018; Walker, 2012) – in Europe, at least, the tendency has been to focus on moral issues rather than on the shortcomings in the market, with regard to approaching financial indebtedness (SOU, 2013:78) – different types of efforts toward counteracting the “morality” of guilt invested in the promise to repay debt in today's economy may be of particular importance. After all, not even the profession of social work has managed to escape the tendency to reduce financial indebtedness to a question of underlying behavioral and cultural problems at the individual level (cf., Callegari et al., 2019; Despard, Chowa, & Hart, 2012; Loke & Hageman, 2013). Furthermore, a key component of debt counselling and other programs that are being considered as effective in minimizing the adverse effects of indebtedness on health, including using alcohol as a self-regulating response to the emotional distress that comes with financial strain (Butler, Dodge, & Faurote, 2010; Serido et al., 2014), concerns the provision of adaptive strategies for coping with debt-related stress (including but not limited to shame and sense of failure) (Turunen & Hiilamo, 2014).

### 4.1 Limitations

The study is entirely based on register data; hence, it lacks self-reported information on confounders that might have influenced the results, for example, self-reported alcohol consumption. However, capturing representative and large groups of individuals with this type of debt issues by means of traditional surveys has, to date, proven to be difficult (Oksanen et al., 2015), which makes register-based studies of the kind presented here important (Thygesen & Ersbøll, 2014). Furthermore, and without downplaying the fact that more studies are needed to fully explore the underlying mechanisms that may be at work in the relationship between economic circumstances and alcohol outcomes, we know from previous research that people with low economic status are more negatively affected by the effect of alcohol, independent of consumption (Bloomfield, 2020; Boyd et al., 2021; Collins, 2016).

A short follow-up period in the study of alcohol-related mortality tends to come with the so-called rare events problem (Eliason, 2014). However, sensitivity tests, excluding one at a time, underlying causes of death from non-alcohol attributable diseases (e.g., fatal alcohol poisonings) and alcohol attributable diseases (e.g., alcoholic liver diseases and alcoholic diseases of the pancreas), respectively, do not suggest that there are some disease specific observations that are driving the results. Furthermore, the study has made use of the BIC as a complementary statistical tool when selecting the models, which has been deemed to be consistent even as the sample size grows infinitely large, at least for the simple regressions models (Vrieze, 2012).

### 4.2 Conclusion

Debt repayment problems have a detrimental impact on alcohol-related death. This effect is statistically independent of other important socio-economic stressors, health (e.g., hospitalization for substance abuse), and demographic characteristics that are not only well-established risk factors for alcohol-related death, but they are also key factors behind financial-indebtedness. Thus, the experience of being registered at an enforcement authority due to unmet financial obligations needs to be treated as an important life event in itself, that is, it is not reducible to the factors that enable people to remain in a financially stable situation.

## Ethics statement

The research project has been approved by the Swedish Ethical Review Authority (reference number: 2017/2227-31/5). The large-scale nature of the register study made the question of informed consent non-applicable. All the data are stored at Statistics Sweden and have been made available to the author via its micro-data Online Access (MONA) system.

## Funding

The research was funded by the 10.13039/501100006636Swedish Research Council for Health, Working Life and Welfare (Grant: dnr 2017-00083).

## Author statement

This is a single-author article.

## Declaration of competing interest

The authors declare no conflict of interest.

## Figures and Tables

**Table A.1:** Codes in the Swedish version of 10^th^ revision of the International Statistical Classification of Diseases (ICD-10-SE) that the National Board of Health and Welfare recommend the healthcare system in Sweden (outpatient and inpatient care) to use when classifying use and abuse of alcohol at a national, regional, and local level.

ICD-10-SE codes	Descriptions
E24.4	Alcohol-induced pseudo-Cushing syndrome
F10.0	Mental and behavioural disorders due to use of alcohol: Acute intoxication
F10.1	Mental and behavioural disorders due to use of alcohol: Harmful use
F10.2	Mental and behavioural disorders due to use of alcohol: Dependence syndrome
F10.2A	Alcohol dependence syndrome with physiological signs of dependence
F10.2B	Alcohol dependence syndrome without physiological signs of dependence
F10.2X	Alcohol dependence syndrome, unspecified
F10.3	Mental and behavioural disorders due to use of alcohol: Withdrawal state
F10.4	Mental and behavioural disorders due to use of alcohol: Withdrawal state with delirium
F10.5	Mental and behavioural disorders due to use of alcohol: Psychotic disorder
F10.6	Mental and behavioural disorders due to use of alcohol: Amnesic syndrome
F10.7	Mental and behavioural disorders due to use of alcohol: Residual and late-onset psychotic disorder
F10.7A	Alcohol dementia
F10.7W	Alcohol-induced mental and behavioural disorders as residual or late-onset psychotic disorder, except alcohol dementia
F10.8	Mental and behavioural disorders due to use of alcohol: Other mental and behavioural disorders
F10.9	Mental and behavioural disorders due to use of alcohol: Unspecified mental and behavioural disorder
G31.2	Degeneration of nervous system due to alcohol
G62.1	Alcoholic polyneuropathy
G72.1	Alcoholic myopathy
I42.6	Alcoholic cardiomyopathy
K29.2	Alcoholic gastritis
K70.0	Alcoholic fatty liver
K70.1	Alcoholic hepatitis
K70.2	Alcoholic fibrosis and sclerosis of liver
K70.3	Alcoholic cirrhosis of liver
K70.4	Alcoholic hepatic failure
K70.9	Alcoholic liver disease, unspecified
K85.2	Alcohol-induced acute pancreatitis
K86.0	Alcohol-induced chronic pancreatitis
O35.4	Maternal care for (suspected) damage to fetus from alcohol
P04.3	Fetus and newborn affected by maternal use of alcohol
Q86.0	Fetal alcohol syndrome (dysmorphic)
R78.0	Finding of alcohol in blood
T51.0	Toxic effect: Ethanol
Y90	Evidence of alcohol involvement determined by blood alcohol level
Y90.0	Blood alcohol level of less than 20 mg/100 ml
Y90.1	Blood alcohol level of 20–39 mg/100 ml
Y90.2	Blood alcohol level of 40–59 mg/100 ml
Y90.3	Blood alcohol level of 60–79 mg/100 ml
Y90.4	Blood alcohol level of 80–99 mg/100 ml
Y90.5	Blood alcohol level of 100–119 mg/100 ml
Y90.6	Blood alcohol level of 120–199 mg/100 ml
Y90.7	Blood alcohol level of 200–239 mg/100 ml
Y90.8	Blood alcohol level of 240 mg/100 ml or more
Y90.9	Presence of alcohol in blood, level not specified
Y91	Evidence of alcohol involvement determined by level of intoxication
Y91.0	Mild alcohol intoxication
Y91.1	Moderate alcohol intoxication
Y91.2	Severe alcohol intoxication
Y91.3	Very severe alcohol intoxication
Y91.9	Alcohol involvement, not otherwise specified
Z71.4	Alcohol abuse counselling and surveillance
Z72.1	Alcohol use

**Table B.1:** BIC-values for the models (2, 3, and 4) presented in Table 2 with added interaction terms.

Multiplicative Models	BIC-value
**Model 2 in**Table 2**+Interaction terms**	
Debt registered at the Enforcement Authority*Age	2903.7
Debt registered at the Enforcement Authority*Gender	2901.6
Debt registered at the Enforcement Authority* Single-person household	2904.3
Debt registered at the Enforcement Authority*Age +	
Debt registered at the Enforcement Authority*Gender +	
Debt registered at the Enforcement Authority* Single-person household	2926.1
**Model 3 in**Table 2**+ Interaction terms**	
Debt registered at the Enforcement Authority* Unemployment	2852.3
Debt registered at the Enforcement Authority* Social welfare recipiency	2853.6
Debt registered at the Enforcement Authority* Disposable income	2855.4
Debt registered at the Enforcement Authority* Education	2868.2
Debt registered at the Enforcement Authority* Criminality	2858.9
Debt registered at the Enforcement Authority* Unemployment +	
Debt registered at the Enforcement Authority* Social welfare recipiency +	
Debt registered at the Enforcement Authority* Disposable income +	
Debt registered at the Enforcement Authority* Education +	
Debt registered at the Enforcement Authority* Criminality	2909.8
**Model 4 in**Table 2**+ Interaction terms**	
Debt registered at the Enforcement Authority* Mood disorder	2761.7
Debt registered at the Enforcement Authority* Mental and behavioral disorders due to psychoactive substance use	2755.2
Debt registered at the Enforcement Authority* Digestive diseases	2764.3
Debt registered at the Enforcement Authority* Mood disorder +	
Debt registered at the Enforcement Authority* Mental and behavioral disorders due to psychoactive substance use +	
Debt registered at the Enforcement Authority* Digestive diseases	2779.6
Total study population (n)	n = 309,689
